# A Loss to the Seamen's Hospital

**Published:** 1912-12-07

**Authors:** 


### A Loss to the Seamen's Hospital.

THE LATE MR. JOHNSON SMITH.

THERE died at Kenley, Surrey, on November 26, William Johnson Smith, F.R.C.S., aged seventy-three. Practically the whole of Mr. Smith's career was in association with the Seamen's Hospital Society. He took the Fellowship from King's in 1866, and was elected assistant to the surgeon (the house surgeon of the period) on board the *Dreadnought*. The hospital was then maintained afloat aboard a man-o'-war placed at the disposal of the Charity by the Admiralty. The hospital was removed ashore to the infirmary of Greenwich Hospital the following year, and with it came Mr. Smith. His death removes the last remaining member of the medical staff who served afloat. Mr. Smith was appointed resident surgeon in 1871 and principal medical officer in 1875. Although taking an active part in the administrative work of his department, he ranked with the members of the hon. medical staff and shared with them the surgical beds in the hospital. In consequence of failing health he retired on pension in 1906, and was at the same time elected a consulting surgeon. Mr. Smith's service extended over the most active period of the Society's existence. Not only did he see the hospital moved on shore to a commodious and properly arranged building, but he assisted in the establishment of the branch hospital of the Society in the Albert and Victoria Docks in 1890, and the dispensaries in the East India Dock Road and Gravesend. He also saw the opening of the London School of Tropical Medicine, and served as surgeon to the hospital to which that school is attached. There are many surgeons of to-day who owe much to Mr. Smith's instruction. He organised the highly successful classes for operative surgery, and was the principal teacher for upwards of twenty years. At one time he was probably known to a larger number of the surgical members of his profession than any other practitioner of his day. He was a linguist of no mean order, and translated many professional works, elaborating the subject in a preface or foreword, and adding many notes and comments. The chief interest of his leisure lay in his library, in which he spent many hours, as befitted the well-read and cultured gentleman that he was. He leaves a widow and one son.

